# Differential Metabolism of Glycerol Based on Oral versus Intravenous Administration in Humans

**DOI:** 10.3390/metabo12100890

**Published:** 2022-09-22

**Authors:** Ankit Shah, Yujue Wang, Fredric E. Wondisford

**Affiliations:** 1 Division of Endocrinology, Metabolism, and Nutrition, Department of Medicine, Robert Wood Johnson Medical School, Rutgers University, New Brunswick, NJ 08901, USA; 2 Rutgers Cancer Institute of New Jersey, New Brunswick, NJ 08901, USA

**Keywords:** gluconeogenesis, glycerol, lactate, mass spectrometry, carbon flux, Cori cycle, portal metabolism

## Abstract

Glycerol can be metabolized to glucose via gluconeogenesis or lactate via glycolysis. It is unknown if glycerol is metabolized similarly in the portal and systemic circulations in humans. Eight metabolically healthy overnight-fasted individuals received equimolar amounts of ^13^C_3_-glycerol orally and intravenously on two separate occasions with serial blood draws over four hours. Serum samples underwent liquid chromatography–mass spectrometry analysis. Oral ^13^C_3_-glycerol administration led to higher average serum glucose enrichment than intravenous administration (5.02 ± 1.43 versus 4.07 ± 0.79%, *p* = 0.009). In contrast, intravenous ^13^C_3_-glycerol administration yielded higher average serum lactate enrichment than oral administration (5.67 ± 0.80 versus 4.85 ± 1.30%, *p* = 0.032). Peak serum glucose enrichment was also higher with oral administration (9.37 ± 2.93 versus 7.12 ± 1.28%, *p* = 0.010). Glycerol metabolism across the portal and systemic circulations is not congruent. Orally administered labeled glycerol led to greater labeled glucose production, while intravenously administration yielded greater lactate production. These data support direct glycerol to lactate conversion in humans.

## 1. Introduction

Lipolysis of triglycerides in adipocytes releases glycerol, a three-carbon molecule, into circulation. Glycerol is a well-known contributor to gluconeogenesis in the liver. We also recently showed in mice [1] and humans [2] that glycerol can bypass glucose and convert directly to lactate, a glycolytic intermediate.

The site(s) of direct glycerol-to-lactate metabolism remains unclear. The liver can potentially convert glycerol to glucose or lactate as it has a high expression of glycerol kinase, which converts glycerol to glycerol-3-phosphate [3]. In contrast, tissues such as the thyroid, adrenal, lung, skeletal muscle, and heart expressing glycerol kinase but not gluconeogenic enzymes allow glycerol metabolism to lactate [4]. Additionally, tissues, including skin, intestines, skeletal muscle, and lungs, express aquaporin channels that enable glycerol transport across cell membranes [5,6]. Despite this distribution of glycerol transporters and glycerol kinase, it is unknown if direct glycerol-to-lactate metabolism occurs in non-hepatic tissues, particularly in humans. 

In this study, we studied the potential of non-hepatic glycerol metabolism in humans by assessing if the route of administration affects glycerol metabolism using a ^13^C_3_-glycerol tracer. This isotope tracer is chemically identical to the endogenous glycerol in the human body but has additional neutrons in its atomic nuclei, giving it a higher molecular mass. The metabolism of the “heavy” glycerol molecule to downstream metabolites can be detected using mass spectrometry, which is sensitive enough to discern mass differences between labeled and unlabeled carbons. We hypothesized that orally administered labeled glycerol would lead to increased labeled glucose production due to hepatic first-pass metabolism. Conversely, we hypothesized that intravenously administered labeled glycerol would lead to greater labeled lactate production as non-hepatic tissues would have a greater opportunity to metabolize the circulating glycerol initially. 

## 2. Materials and Methods

### 2.1. Human Participants

All subjects underwent informed consent, and the Rutgers University Institutional Review Board approved all study procedures. Inclusion criteria included general good health and no evidence of diabetes mellitus as indicated by the American Diabetes Association diagnostic criteria [7]. Exclusion criteria included chronic medical conditions that may affect glucose metabolism, including active malignancy, kidney disease, liver dysfunction, alcoholism, pancreatitis, and current pregnancy/lactation, as well as the use of medications that may affect glucose metabolism, including glucocorticoids.

### 2.2. Experimental Protocol

Study subjects came in for two separate study visits, which occurred in random order and were separated by at least one week to allow for metabolic clearance of the tracer. On the evening before each study visit, subjects completed dinner by 8 PM at home and began an overnight fast. Subjects arrived at the Clinical Research Center at Robert Wood Johnson Medical School (RWJMS) at 8 AM.

For one study visit, at 9 AM (time = 0 min), study subjects ingested ^13^C_3_-glycerol (Sigma Aldrich 660701, St. Louis, MO, USA) at 50 mg/kg, diluted in sterile water to 0.1 g/mL; this dose comes from prior studies that gave humans a ^13^C_3_-glycerol oral bolus [8,9,10,11]. Subjects consumed the solution within two minutes. Blood samples were collected at times −10, 2, 5, 10, 15, 30, 60, 90, 120, 150, 180, and 240 min from an intravenous catheter placed in the arm. After the final blood draw, the nursing staff removed the intravenous catheters. Blood samples were collected into a serum separator tube, allowed to clot at room temperature (RT) for 30 min, and then centrifuged at 900× *g* for 10 min at RT. Serum was stored in a −80 °C freezer until liquid chromatography–mass spectrometry (LC-MS) analysis.

For the other study visit, subjects intravenously received ^13^C_3_-glycerol at 50 mg/kg, diluted in sterile normal saline at 0.1 g/mL at 9AM (time = 0 min). Subjects received intravenous catheter placements on each arm, one for tracer infusion and the second for blood collection. Blood collection procedures were similar to the visit involving oral administration.

### 2.3. Glycerol and Glucose Derivatization

Given the poor ionization of glycerol and glucose, an enzymatic derivatization was required for detection in LC-MS [12]. Serum samples were added into a 10× volume of reaction buffer containing 25 mM Tris (pH 8.0), 10 mM Mg^2+^, 50 mM NaCl, 5 mM ATP, 2 U/mL glycerol kinase (Sigma-Aldrich G6278, St. Louis, MO, USA), and 2 U/mL glucose kinase (Sigma-Aldrich H4502) and incubated for 10 min at RT. Serum samples were then quenched with −20 °C 40:40:20 methanol:acetonitrile:water solution with 0.5% formic acid followed by incubation at RT for 5 min. The mixture was neutralized with 15% NH_4_HCO_3_ solution and centrifuged at 16,000× *g* for 10 min. The supernatant was transferred to a clean tube for LC-MS analysis. The same reaction was also performed in a blank tube to remove background signals.

### 2.4. LC-MS Analysis

Prepared samples underwent LC-MS analysis, as previously described [13]. Briefly, liquid chromatography separation occurs on Waters Xbridge BEH Amide columns using solvent A (20 mM ammonium acetate + 20 mM ammonium hydroxide in 95:5 water:acetonitrile, pH 9.4) and solvent B (20 mM ammonium acetate + 20 mM ammonium hydroxide in 20:80 water:acetonitrile, pH 9.4). The flow rate was 300 μL/min with the column temperature set at 25 °C. Mass spectrometry scans were obtained under negative polarity in a stand-alone orbitrap mass spectrometer (ThermoScientific Q Exactive Plus MS, Waltham, MA, USA). Analysis was performed on a blank tube to account for background signals. Data were analyzed using the MAVEN software suite [14]. The natural isotope abundances were corrected using AccuCor [15]. Total pool sizes for each substrate were determined by adding internal standards into the serum sample at concentrations of 0.1 mM D_8_-glycerol (Cambridge Isotope DLM-558. Tewksbury, MA, USA), 1 mM D_3_-lactate (Cambridge Isotope DLM-9071), and 5 mM ^13^C_6_D_7_-glucose (Cambridge Isotope CDLM-3813).

### 2.5. Calculations

Carbon enrichment data was calculated as atom percent excess with the formula [(^13^C)/(^12^C+^13^C)] × 100. Data are reported as mean ± SD, except in figures, where they are reported as mean ± SE. Areas under the curve (AUC) were calculated using the trapezoidal method [16].

### 2.6. Statistical Analysis

A two-tailed paired t-test was used to compare data gathered between the two study visits. Data normality was tested using the Shapiro–Wilk test, and the Wilcoxon signed-rank test was used for nonparametric data. All analyses were performed on SPSS (Version 27.0, Armonk, NY, USA) with significance set at *p* < 0.05.

## 3. Results

### 3.1. Subject Characteristics

As shown in Table 1, study participants had no evidence of metabolic abnormalities, including hyperglycemia, hyperlipidemia, kidney disease, or liver dysfunction.

### 3.2. Enrichment Data and Total Pool Size

**Figure 1:**
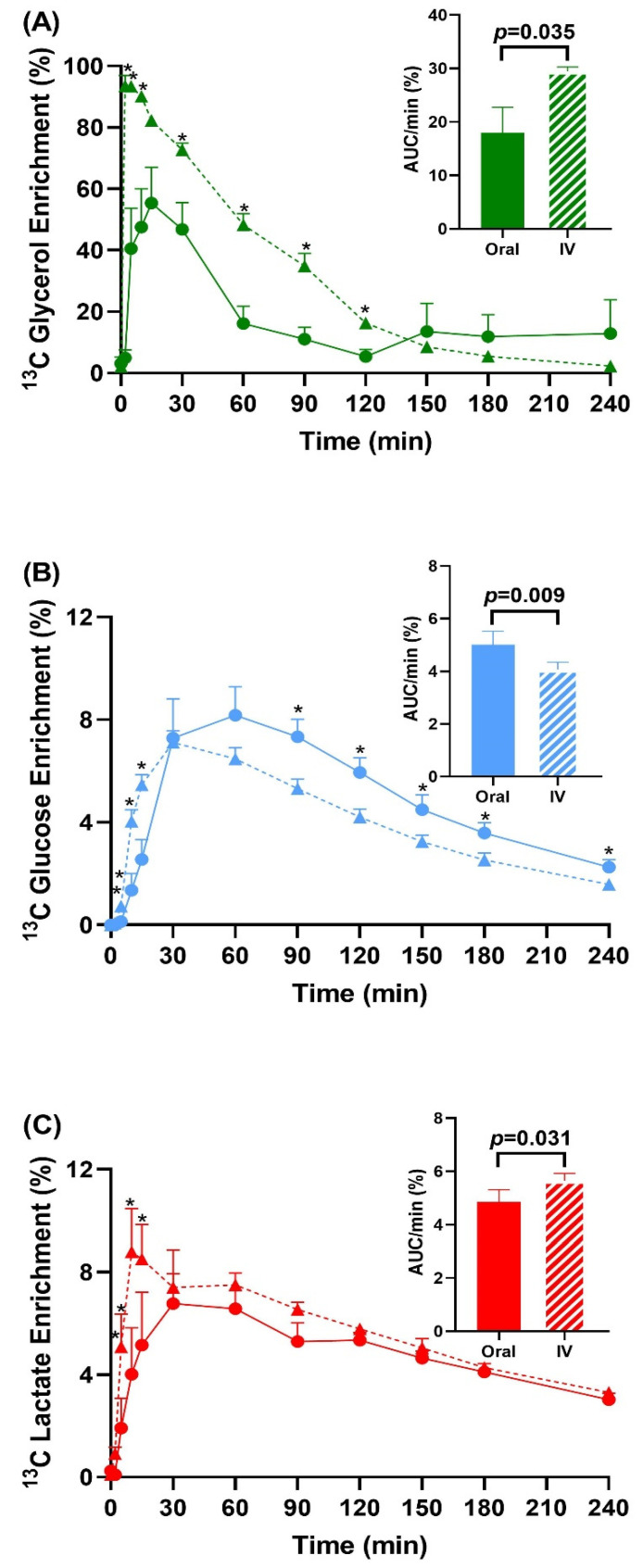
^13^C-enrichment data of glycerol (**A**), glucose (**B**), and lactate (**C**) after oral (solid) and intravenous (dashed, IV) ^13^C_3_-glycerol administration. Inset graphs have the area under the curve (AUC) data. * *p* < 0.05 via paired t-test or Wilcoxon sign-rank test between oral and intravenous administration. *n* = 8.

Throughout the four-hour study period, average systemic glycerol ^13^C enrichment, measured by area under the curve (AUC) per minute, was higher with intravenous administration than with oral administration (29.47 ± 2.26 versus 18.02 ± 13.27%, *p* = 0.035, Figure 1A). Glucose enrichment was higher with oral versus intravenous administration (5.02 ± 1.43 versus 4.07 ± 0.79%, *p* = 0.009, Figure 1B). In contrast, lactate enrichment was higher with intravenous administration versus oral administration (5.67 ± 0.80 versus 4.85 ± 1.30%, *p* = 0.031, Figure 1C).

**Figure 2:**
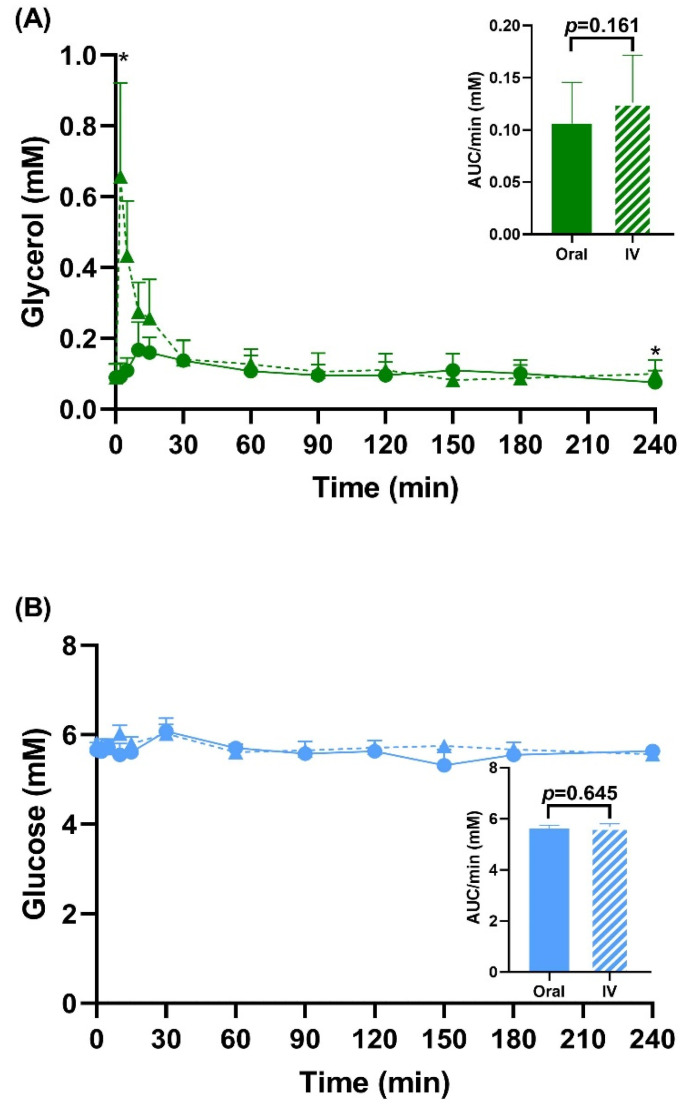

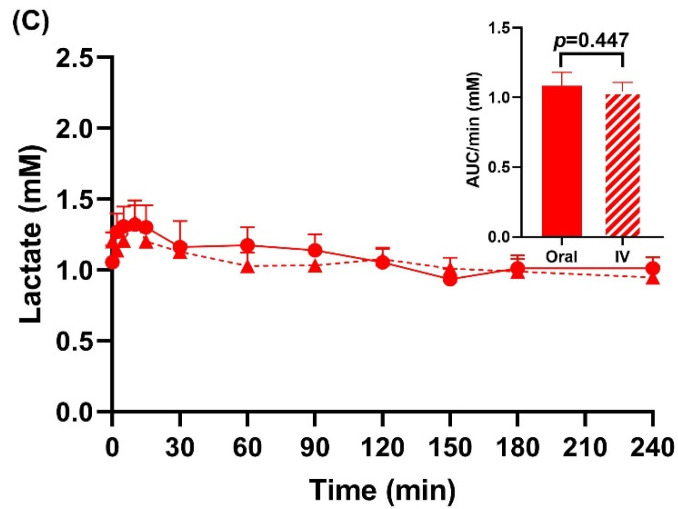
Total circulating concentrations of glycerol (**A**), glucose (**B**), and lactate (**C**) for oral (solid) and intravenous (dashed, IV) ^13^C_3_-glycerol administration. Inset graphs have the area under the curve (AUC) data. * *p* < 0.05 via paired t-test or Wilcoxon sign-rank test between oral and intravenous administration. *n* = 8.

Figure 2 shows the total circulating concentrations of glycerol, glucose, and lactate after oral and intravenous administration of ^13^C_3_-glycerol. Serum glycerol concentrations were elevated after intravenous administration versus oral administration, but only at the 2 min (0.657 ± 0.748 versus 0.091 ± 0.108 mM, *p* = 0.017) and 240 min time points (0.100 ± 0.110 versus 0.075 ± 0.095 mM, *p* = 0.028). Total glucose and lactate levels were unchanged during the four-hour study period and were not different between the two administration modes. 

### 3.3. Peak Glucose and Lactate Enrichment Data

As seen in Supplemental Figure S1, during oral ^13^C_3_-glycerol administration, the time to peak enrichment for glucose and lactate were comparable, 60.00 ± 27.77 versus 61.88 ± 42.76 min, *p* = 1.000. For intravenous ^13^C_3_-glycerol administration, time to peak enrichment for glucose and lactate was respectively 33.75 ± 10.61 and 26.88 ± 30.81 min, *p* = 0.566.

**Figure 3:**
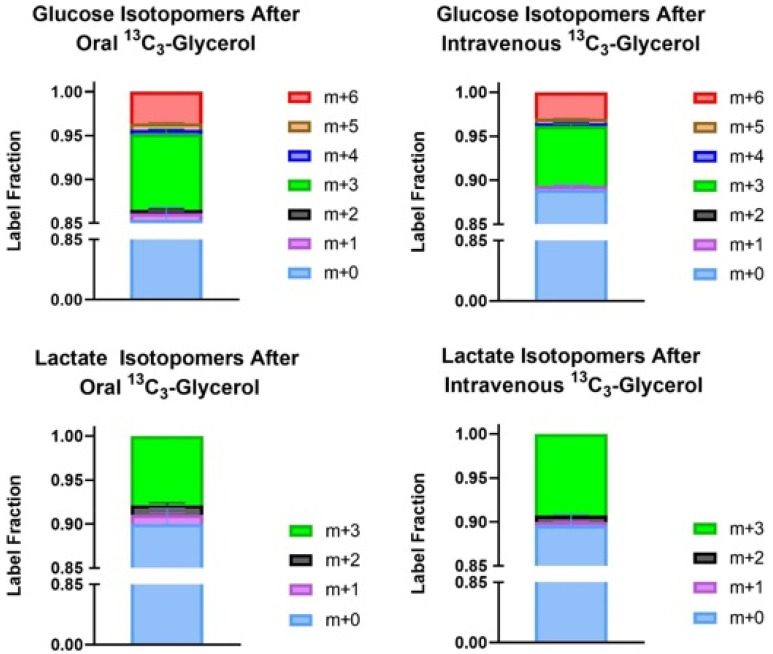
Isotopomer distribution of carbon-labeled glucose and lactate during peak enrichment of each metabolite after ^13^C_3_-glycerol administration. *n* = 8.

Peak glucose enrichment was higher with oral ^13^C_3_-glycerol administration versus intravenous (9.37 ± 2.93 versus 7.12 ± 1.28%, *p* = 0.010). Peak lactate enrichment was comparable between the oral and intravenous administration, respectively, 8.92 ± 4.50 and 9.89 ± 3.45%, *p* = 0.263. Figure 3 shows the isotopomer distribution of carbon-labeled glucose and lactate molecules for each subject at the time of peak enrichment for each respective metabolite during oral and intravenous administration. Most labeled glucose produced during oral and intravenous administration was of m + 3 distribution, followed by m + 6, and then m + 1. The majority of labeled lactate produced was of m + 3 distribution.

## 4. Discussion

**Figure 4:**
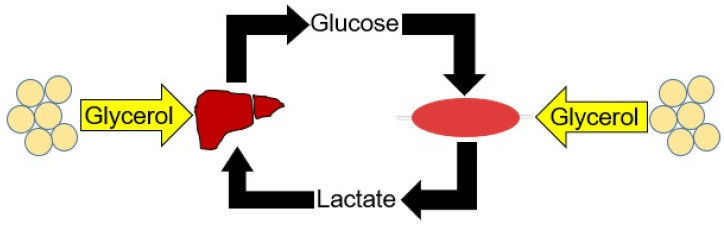
Glycerol from adipose tissue can contribute carbons to the Cori cycle, shown in black arrows, in two ways. The liver converts glycerol to glucose while peripheral organs, represented schematically as skeletal muscle, can convert the molecule to lactate.

The present study assessed glycerol metabolism in metabolically healthy humans based on oral and intravenous administration as a surrogate for studying portal and systemic metabolism. Here, we show disparate glycerol carbon fluxes across the two parallel circulatory beds (Figure 4). Orally administered glycerol leads to more glucose production, while intravenous administration produces more lactate.

Enhanced glycerol-to-glucose metabolism in the portal circulation has implications for type 2 diabetes pathogenesis. In T2DM, hepatic gluconeogenesis rates are elevated up to 40% [17], and underlying increases in adiposity and insulin resistance increase glycerol release into circulation [18,19,20]. Further, Gastaldelli et al. found that increased visceral adiposity correlates with enhanced gluconeogenic activity in a cohort of patients with T2DM [21]. Compared to subcutaneous fat, visceral fat has higher glycerol release, drains directly into the portal vein, and poses a higher risk for metabolic syndrome [22]. Thus, direct glycerol drainage from visceral fat into the portal vein may lead to increased hepatic glucose production.

Glycerol is a common food additive used in processed meats, dairy products, sweetened beverages, and confectionaries [23]. Its sweet taste and smooth texture make it a sugar substitute and prolong product shelf life. Additionally, ingested dietary fat contains glycerol. Thus, overall glycerol intake may have an underrecognized impact on prandial glucose levels, particularly in patients with diabetes who take mealtime insulin for glucose control. These patients may need to account for their meals’ glycerol content alongside traditional carbohydrates.

Figure 1A shows that less orally administered glycerol reaches systemic circulation than intravenous administration, suggesting rapid glycerol metabolism. The intestines and/or gut microbiota may metabolize orally administered glycerol before the molecule reaches the liver. We did not collect blood samples from the portal vein, as this would require technically invasive catheter cannulations that would significantly increase risks to our subjects. However, Previs et al. showed that glycerol concentrations are 20% lower in the portal vein of dogs compared to arterial circulation and provided evidence for increased glycerol uptake and release by the intestinal tissue [24]. Thus, the rate and fate of metabolic flux of glycerol may differ across the portal and systemic circulations, though further confirmatory studies using animal and in vitro models are needed.

The Cori cycle is an important source of serum lactate from circulating glucose. However, as shown in Supplemental Figure S1B, the Cori cycle is unlikely to be the only source of increased labeled lactate after intravenous ^13^C_3_-glycerol admission, as lactate enrichment is significantly higher than glucose enrichment for most time points. If the carbon flow were only glycerol to glucose to lactate, lactate enrichment could only be as high as glucose enrichment. This enhanced lactate production with intravenous labeled glycerol administration suggests direct glycerol-to-lactate metabolism by non-hepatic tissues, a previously unknown fate of glycerol.

During intravenous administration, we can expect the liver to see about 25% of the infused glycerol based on the percentage of cardiac output to the liver [25]. We assume that gastrointestinal and hepatic tissues initially receive 100% of the glycerol compound during oral administration. Additionally, the labeled lactate molecules are mainly m + 3 isotopomers, indicating that the glycerol carbon backbone is preserved in the newly made lactate. Based on this experiment, it is unknown which non-hepatic tissues metabolize circulating glycerol directly to lactate. To decipher this, one can study glycerol metabolism using different tissues and organoids in an in vitro setting. Alternatively, in vivo methods could include direct arterial and venous cannulations with ^13^C_3_-glycerol administration across different organs.

Glycerol to lactate conversion in non-hepatic tissues also has clinical relevance. In Japan, the drug Glycereb^TM^, a combination of fructose and glycerol, treats elevated intracranial pressure by working as an osmotic diuretic agent. Studies showed increased serum lactate levels after intravenous infusion of Glycereb^TM^ compared to mannitol in critically ill patients [26]. Fructose was thought to be the source of lactate elevation, as fructose can enter glycolysis and contribute to lactate production [27]. However, our study suggests that intravenous glycerol could also contribute to this lactate elevation, particularly as the molar concentrations of glycerol to fructose in Glycereb^TM^ are four to one. Increased lactate levels in a patient can be a marker of sepsis or impaired tissue perfusion, affecting clinical management. Physicians must be mindful of potential lactate elevation from the infusion of various organic compounds.

## 5. Conclusions

In summary, orally administered labeled glycerol leads to greater labeled glucose production, and intravenously administered labeled glycerol leads to greater labeled lactate production in humans. Future studies should assess if other substrates are processed differently based on the administration route to differentiate metabolism across the portal and systemic circulations.

## Figures and Tables

**Table 1:** Subject characteristics. Data are mean ± SD.

	Value	Reference
Gender (M/F)	4/4	
Age (years)	29.1 ± 7.5	
Weight (kg)	69.9 ± 8.51	
BMI (kg/m^2^)	22.8 ± 2.78	18.5–24.9
Fasting glucose (mmol/L)	4.72 ± 0.32	3.6–5.6
Creatinine (mg/dL)	0.86 ± 0.19	0.65–1.35
Total cholesterol (mg/dL)	195.3 ± 34.7	<200
Triglycerides (mg/dL)	82.4 ± 22.6	<150
High-density lipoprotein cholesterol (mg/dL)	56.0 ± 16.8	>40
Low-density lipoprotein cholesterol (mg/dL)	122.9 ± 35.0	<160
Aspartate aminotransferase level (IU/L)	21.5 ± 8.00	10–40
Alanine aminotransferase level (IU/L)	16.9 ± 5.94	9–46

## Data Availability

The data presented in this study are available on request from the corresponding author.

## Supplementary Materials

The following supporting information can be downloaded at: https://www.mdpi.com/article/10.3390/metabo12100890/s1, Figure S1. ^13^C-enrichment data of glucose (blue) and lactate (red) after oral (A) and intravenous (B) ^13^C3-glycerol administration. Inset graphs with the area under the curve (AUC) data. * *p* < 0.05 via paired t-test or Wilcoxon sign-rank test between oral and intravenous administration. *n* = 8.
