# Supplementary material for: Differential Metabolism of Glycerol Based on Oral versus Intravenous Administration in Humans
Source: Metabolites. 2022 Sep 22;12(10):890. doi: 10.3390/metabo12100890 (PMC9611849; doi:10.3390/metabo12100890)
Supplement: Supplementary file 1 [file metabolites-12-00890-s001.zip › metabolites-1915052-supplementary.pdf]

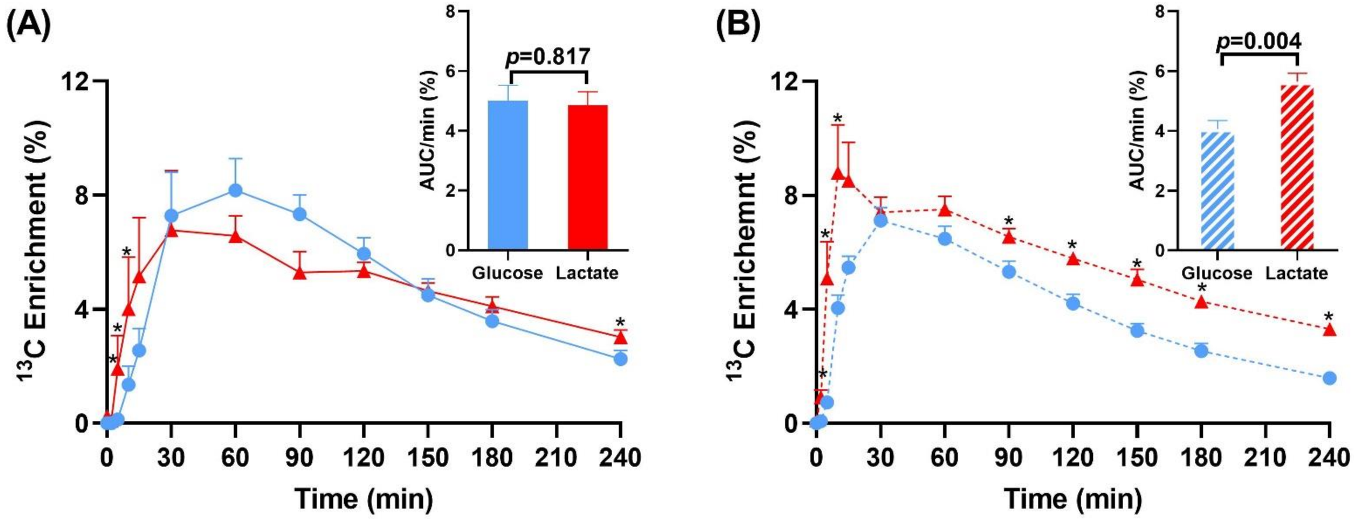

Supplementary Figure S1. ^{13}C-enrichment data of glucose (blue) and lactate (red) after oral (A) and intravenous (B) $^{13}C_3$-glycerol administration. Inset graphs with the area under the curve (AUC) data. *$p<0.05$ via paired t-test or Wilcoxon sign-rank test between oral and intravenous administration. n=8.
